# Cross-Cultural Applicability of Organizational Stressor Indicator for Sport Performers Questionnaire in Ghana Using Structural Equation Modeling Approach

**DOI:** 10.3389/fpsyg.2021.772184

**Published:** 2021-12-13

**Authors:** Medina Srem-Sai, Frank Quansah, James Boadu Frimpong, John Elvis Hagan, Thomas Schack

**Affiliations:** ^1^Department of Health, Physical Education, Recreation and Sports, University of Education, Winneba, Ghana; ^2^Department of Educational Foundations, University of Education, Winneba, Ghana; ^3^Department of Health, Physical Education and Recreation, University of Cape Coast, Cape Coast, Ghana; ^4^Neurocognition and Action-Biomechanics-Research Group, Faculty of Psychology and Sports Science, Bielefeld University, Bielefeld, Germany

**Keywords:** cross-cultural, Ghana, organizational stressor, Organizational Stressor Indicator for Sport Performers (OSI-SP), structural equational modeling (SEM)

## Abstract

The purpose of this study was to examine the cross-cultural validity of the Organizational Stressor Indicator for Sport Performers (OSI-SP) scale by investigating its psychometric properties with Ghanaian footballers. The study particularly sought to assess in the Ghanaian context, 1, the convergence validity and reliability of the OSI-SP scale, 2, the discriminant validity of the OSI-SP scale to understand the applicability of its factor structure, and 3, whether the OSI-SP hypothesized model fits the data collected within the study context. The intensity aspect of the OSI-SP questionnaire was administered to 424 Ghana Premier League (GPL) male footballers who took part in the 2020/2021 season. Quality control strategies were put in place to ensure consistency across interpreters and as well improve the validity of the data. The results from a multi-factor first-order confirmatory factor analysis showed some level of convergence validity of the OSI-SP scale in the Ghanaian context using football players. Out of the 23 items on the original scale, 20 met the factor loadings criterion. In assessing the discriminant validity of the OSI-SP scale using Heterotrait-monotrait (HTMT), 50% of the correlation ratios met the criterion for the original 23-item instrument. Comparing the new model (with the 20-items) with the original model (with 23-items) using the Akaike Information Criterion (AIC) value, the model fit indices for the modified model (20-items) appeared better than the original model with 23-items. Generally, there was minimal support for the applicability of the OSI-SP instrument across the sample of Ghanaian footballers. The implications of these findings are discussed in detail.

## Introduction

Organizational stressor, defined as “an ongoing transaction between an individual and the environmental demands associated primarily and directly with the organization within which he or she is operating” (Fletcher et al., 2006, p. 329), has emerged as a key psychological construct in sport performers’ mental preparations before and during competitions (Fletcher et al., 2006; Fletcher and Wagstaff, 2009; Woodman and Hardy, 2001). Existing scholarly investigations in organizational stressors originated from research in organizational behavior where scholars discovered that when employees are overwhelmed with their perceived workloads such that their resources to cope with such workloads are inadequate, they experience episodes of stress (Sirom, 1982). The organizational stress literature indicates that organizational stressors generally comprise workloads, performance in sales, conflicts among and between parties, as well as constraints in an organization (Spector and Jex, 1998). Similarly, organizational stress has been found to have an association with burnout and absenteeism among employees (Westman and Etzion, 2001), including motivation as well as performance (LePine et al., 2004), mental health and cardiovascular health complications (Cooper and Marshall, 2013). Within sport, organizational stressors have also been linked with athletes’ burnout (Tabei et al., 2012; Arnold et al., 2013), negative emotions and affect (Fletcher et al., 2012), compromised athletic or sports performance (Fletcher et al., 2006; Lu et al., 2012; Fletcher and Arnold, 2016), and dysfunctional health and well-being (DiBartolo and Shaffer, 2002). Given that these probable health implications are somewhat alarming due to their prevalence and the high proportion of organizational demands in sport performers, continuously examining their influence with accurate measurement indicators on individuals still remains relevant.

One instrument that has gained considerable attention in the measurement of organizational stressors in sport is the “Organizational Stressor Indicator for Sport Performers (OSI-SP)” developed and validated by Arnold et al. (2013). The OSI-SP is a 23-item scale with five domains used for measuring the frequency, intensity and duration of organizational stressors in the context of sports (Arnold et al., 2013). The domains of the OSI-SP are goals and development (captures organizational stressors that have a link with individuals’ feedback, advancement and transition in their sport); logistics and operations (entails organizational stressors that are related with the arrangement and application of processes necessary for training and or competitive events); team and culture (indicates organizational stressors that are related with the attitudes and behaviors that members exhibit in the team); coaching (consists of organizational stressors that are related to the personality and interpersonal attributes of the coach) and selection (covers the organizational stressors that are related with how players or performers of a sport are chosen to be part of a team or forthcoming competitive events) (Arnold et al., 2013).

Several scholars have employed the OSI-SP to examine the organizational stressors of various sport performers in different cultural contexts and found different results (Fletcher et al., 2012; Lu et al., 2012; Tabei et al., 2012; Arnold et al., 2017a). For example, Arnold et al. (2017a) employed the OSI-SP on a sample of 414 sport performers across a number of sport disciplines in the United Kingdom and found a positive linkage among goals and development stressors, team and culture stressors, and negative affect. Despite gaining much understanding on the OSI-SP by various scholars in differing contextual setups, only a few have cross-culturally validated the OSI-SP (Arnold et al., 2013, 2016, 2017b; Liu et al., 2018), with empirical findings on its applicability in other geographical boundaries still being inconclusive. Although some scholars found that the OSI-SP had adequate applicability on Taiwanese (Liu et al., 2018), British and Malaysian (Arnold et al., 2017b) and British (Arnold et al., 2013) athletes, the certainty that the OSI-SP would be applicable in other cross-cultural contexts has also been questioned. For example, Arnold et al. (2017b) investigated the measurement invariance of the OSI-SP among a sample of English, Malaysian, and Chinese athletes. Findings showed that the Chinese data failed to meet all the standard criterion values of measurement invariance and as well did not support the 5-factor 23-item structure of OSI-SP. Other results revealed that the Chinese sample comparatively recorded lower internal consistency values on the goals and development subscale. Overall, Arnold and partners reported that the Chinese data did not fit the model, citing cultural variations as potential reasons accounting for the findings. Similarly, Liu et al. (2018) examined the psychometric properties of the OSI-SP with Taiwanese athletes through four interrelated studies. The results revealed that the 5-factor, 16-item Chinese OSI-SP had adequate factor structure, measurement invariance, criterion validity, and reliabilities. However, the original OSI-SP version was shortened by dropping seven items due to reported cultural and contextual norms.

Of particular interest for cross-cultural comparison is the OSI-SP data from athlete samples drawn from East Asian countries like China, Malaysia and Taiwan, where cultural issues are quite pervasive (Arnold et al., 2017b; Liu et al., 2018). These societies are classified predominantly as collectivist cultures (i.e., a belief that the group’s interests supersede those of individuals and that sharing and cooperation are key to the maintenance of a harmonious society) with unique linguistic peculiarities (speak Mandarin, the similar language across them) (Hofstede, 1980; Triandis et al., 1988; Triandis, 1995). Existing cross-cultural studies have already shown that culture strongly impacts one’s values and attitudes, including cognition, emotion, and motivation. According to Markus and Kitayama (1991), cultural norms and values have considerable influence on the construal of self, of others, and the interdependence between self and others.

Like most sub-Saharan countries, Ghana represents another collectivist unique cultural setting similar to East Asia although with its multi-lingual and multi-ethnicity orientations of cultural diversity (Lentz and Nugent, 2000). Specifically, although there is significant linguistic heterogeneity in Ghana with over forty indigenous languages (Obeng, 1997), two languages: Akan and Hausa, in addition to English have emerged as the most important lingua francas in different communicative contexts for varied reasons (e.g., inter-group communication), especially in urban centers (Obeng, 1997; Anyidoho and Dakubu, 2008). Similar to East Asian countries, Ghana like many other sub-Saharan societies has a cultural orientation likened to Confucian philosophy which teaches the indigenes to observe dominant codes such as subservience to authority, upholding social orders, respect for the elderly, parents, and teachers (Kai, 2008; Ames and Rosemont, 2010; Ampiah, 2014; Kamoche et al., 2015; Metz, 2016). Hence, whether an instrument like OSI-SP would yield adequate level of psychometric properties and factor structure has not been tested empirically within the African context. This creates a void in extant literature which this study intends to address. Sue (1999) acknowledged that cross-cultural comparative studies are lacking enough to guarantee the external validity and applicability of their interpretations, theories, and models in psychological research. According to Fletcher and Arnold (2016), the lack of cross-cultural research on this instrument ought to be addressed going forward before the generalization of the OSI-SP can be supported and utilized across diverse populations and cultures.

Therefore, the purpose of this study was to assess the cross-cultural validity of the psychometric properties of the OSI-SP in the Ghanaian context using football players of the Ghanaian Premier League (GPL). The study particularly sought to assess in the Ghanaian context, 1, the convergence validity and reliability of the OSI-SP scale, 2, the discriminant validity of the OSI-SP scale to understand the applicability of its factor structure, and 3, whether the OSI-SP hypothesized model fits the data collected. Such an examination will ascertain whether the OSI-SP: its conceptualization and operationalization would accommodate the peculiarities of cultural diversity (Sue, 1999; Yang and Jowett, 2012; Arnold et al., 2017b). It is only when enough empirical evidence is provided on both the meaning and dimensional structure of organizational stressors and the items comprising the OSI-SP, can cross-cultural comparisons be made across different geographical boundaries (van de Vijver and Leung, 1997; Byrne et al., 2009; Arnold et al., 2017b).

## Materials and Methods

### Study Participants

This study covered a wide geographical area in Ghana because teams were spread from the Northern to Southern, and Eastern to Western parts of Ghana. Four hundred and twenty-four (*n* = 424) male footballers who took part in the 2020/2021 GPL season agreed to participate in this study using census. The ages of these players ranged from 16 years to 31 years (*M*_*age*_ = 22.36; *SD* = 3.53) and years of experiences ranged from one year to 15 years (*M* = 2.69; *SD* = 1.82) with the majority of them reporting below 5 years of experience. For a player to qualify to play in the GPL, he must have a good performance record and no record of indiscipline. About 2.12% (*n* = 9) of the participants had post-secondary school (diploma and bachelor’s degree) qualifications, 143 (33.72%) had secondary school qualifications, and the remaining 64.16% (*n* = 272) indicated they were educated to the Junior high/primary school level. Generally, the majority of the players (74.5%, *n* = 316) reported they were not fluent in the English language (in terms of writing and speaking) and thus, needed assistance during the data collection.

Translation of the items to the various local dialects was not adopted in this research for three reasons. First, pieces of scholarly research in Ghana has consistently revealed that the majority of young persons in Ghana have difficulties in writing, reading and comprehending written information in their local languages (see Owu-Ewie and Edu-Buandoh, 2014; Ackon, 2015; Bronteng et al., 2020; Dew Research, 2020). For example, a study conducted by Dew Research found that about 80% of the Ghanaian youth cannot read and write in their local language. Secondly, preliminary informal information obtained from the participants revealed that the majority of them had challenges with writing, reading and comprehending written information in their local languages. The participants were only fluent in speaking their local language. Lastly, some of the local languages in Ghana do not have well written and/or consistent forms. Even across the same ethnic group, the same language may be spoken and written differently. Using the Fante language, for example, there are several shades of the language, although it is considered as a single language in Ghana (Bronteng et al., 2020). With these reasons, it was quite obvious that the participants would have still needed interpreters even when the items on the instrument are translated. Therefore, interpreters were instead recruited to assist in the data collection.

### Measures

#### Organizational Stressor Indicator for Sport Performers Scale

The intensity dimension of Arnold et al.’s (2013) 23-item OSI-SP was used to determine the organizational stressors encountered by football players as they participated in the 2020/2021 GPL. Initial instructions on the instrument informed participants to demonstrate honesty and openness, and that if any participant represented two or more teams, they should complete the OSI-SP regarding the team they mostly competed for within the season. The OSI-SP has five subscales namely: Team and Culture (4-items; e.g., “my teammates attitudes”), Goals and Development (6-items; e.g., “my goals”), Logistics and Operations (9-items; e.g., “the training or competition venue”), Selection (2-items; e.g., “selection of my team for competition”) and Coaching (2-items; e.g., “the relationship between my coach and I”). For each item, the stem “In the past month, I have experienced pressure associated with.” is provided, to which all participants responded on only the intensity rating scale that ranges from 0 to 5. The scale for intensity was “how demanding was this pressure”? 0 represents no *demand*; 1, very low demand; 2, low demand; 3, moderate demand; 4, high demand; and 5, very high demand. Arnold et al. (2013) developed and validated this instrument in a series of studies. Reported internal consistency values using Cronbach’s alpha coefficient for the intensity scale ranged from 0.71 to 0.83.

### Procedure

Ethical approval was granted from the Institutional Review Board of the University of Cape Coast (IRB-UCC) to conduct the study with reference number UCC/IRB/A/2016/794. The participants for the study were recruited by meeting with club owners, managers, Chief Executive Officers (CEOs), coaches and the players to discuss the purpose and significance of the study. After familiarization, the study participants were informed about their rights to anonymity, withdrawal from the study at any point in time and confidentiality of all responses given. Participants were also informed that the data would be available to only the researchers. Before data was collected, every participant was asked to willingly endorse a consent form after which the study measure was distributed to them to respond. Responding to the instrument lasted for approximately 15–20 min within a period of 3 months for all teams. Participants who could not solely respond to the instrument were carefully guided and helped with the interpretations of the items in their local dialects. Data were collected at the various camps of the teams.

### Quality Control Strategies

Six research assistants were carefully selected and trained to assist in the data collection bearing in mind the geographical locations (and their respective language) of the GPL teams. The assistants were fluent in English, Twi, Fante, Ewe, Ga, Nzema, Bono and Dagbani languages. All the assistants were fluent in more than two languages. For example, the English and Twi languages were common among all the research assistants. Similarly, the assistant who was fluent in Dagbani was also fluent in other languages of the Northern Region indigenes. The research assistants were taken through the purpose of the study and the instrument for the data collection was exposed to the assistants, from one item to another. Particularly, the scale options were extensively discussed with the assistants to ensure that all of them understood the scale points used. On the discussion of items and item response, deliberations were done using the English language as well as the local dialects of the assistants. They were also oriented on the necessary ethical considerations (such as consent, anonymity, confidentiality, privacy, volition, and rapport creation) which they were required to uphold. One of the assistants dropped out of the data collection during the pilot testing phase. Out of the 5 interpreters who assisted in the data collection, one was a graduate associate/assistant in a university in Ghana, with a background in translations. The rest of the interpreters were postgraduate students reading programs related to specific local languages either as major or minor.

A two-stage pilot testing was carried out after the in-house training. In the first pilot testing, five players who were fluent in the Twi language were purposefully selected by the club managers/coaches for the exercise. The first pilot aimed to establish the extent of consistency among the assistants in interpreting the items and response options to the participants. Participants who were fluent in a common language were sampled for this phase of the research. Further, all the assistants took data from each player selected such that each assistant administered five questionnaires (one for each participant). This process generated 25 copies of responded questionnaires. The extent of item/response-interpreter consistency was computed using Generalized Analysis of Variance (GENOVA) analysis (with *player* ***x*** *item* ***x*** *interpreter* design) (Brennan, 2011). A generalizability index (*g*) of 0.83 was obtained which was sufficient to ensure consistency across the interpreters (Creswell, 2012). Further, round table discussions were carried out to address challenges and to improve the consistency among the assistants when taking the data.

The second phase of the pilot involved 5 other players who were fluent in the Ga, Ewe, Nzema, Bono, and Dagbani languages. Each of the five research assistants who were fluent in English and these languages administered the instrument to the participants by interpreting the items and response options to the players. This exercise was monitored by three trained supervisors (some of the supervisors could speak two of the languages) who were fluent in the languages being used. For each of the research assistants, the accuracy of the data gathered was evaluated over 100 by the supervisor. An average score of 78% was achieved. Meanwhile, each research assistant had a detailed discussion with the supervisors on the weaknesses and challenges encountered to improve the quality of data in the main data collection.

### Data Analyses Plan

The data were screened using means, standard deviation, minimum and maximum values, and outlier analysis to examine any abnormalities. Missing data were also checked and no data point was found to be missing. Then, a series of validation statistical procedures were conducted using Analysis of Moment Structures (AMOS) version 21. The multi-factor first-order confirmatory factor analysis, with five constructs and 23-items, was first specified and modeled (Schumacker and Lomax, 2004). An item each from every factor was fixed at 1.0. To assess the extent of convergence of the items, the factor loadings and the average variance extracted (AVE) estimates were utilized. The reliability of the scale was judged using the composite reliability estimate. The discriminant validity analysis was conducted using the Heterotrait-monotrait (HTMT) ratio of correlation. This approach was chosen over other procedures, such as Fornell and Larcker’s (1981) procedure, because the HTMT is more sensitive and powerful in detecting non-discriminant factors (Hensler et al., 2015). To examine whether the hypothesized model fits the data, model fit indices were examined. Following the recommendations of the literature, several fit indices were used to evaluate the adequacy of the produced model(s). These indices considered include chi-square statistics, Goodness of Fit (GFI), Root Mean Square Error of Approximation (RMSEA), Comparative Fit Index (CFI) and Akaike Information Criterion (AIC) (Mueller and Hancock, 2004; Schumacker and Lomax, 2004; Hair et al., 2016; Civelek, 2018).

## Results

The results of this paper were presented as three separate but interrelated sections. In the first part, the analysis on the convergent validity as well as the reliability estimates were presented by reporting on the factor loadings, AVE, and the composite reliability coefficients of the sub-scales. The second part provides a discriminant analysis of the OSI-SP instrument using the HTMT ratio of correlation. The last part of the analysis looked at the model fit indices for the measurement model which shows the extent to which the data fits the model specified. For all the parts, two forms of analysis are presented; (a) analysis of the original scale, (b) analysis after items with low loadings (<0.05) were removed from the model.

### Convergent Validity and Reliability

**FIGURE 1:**
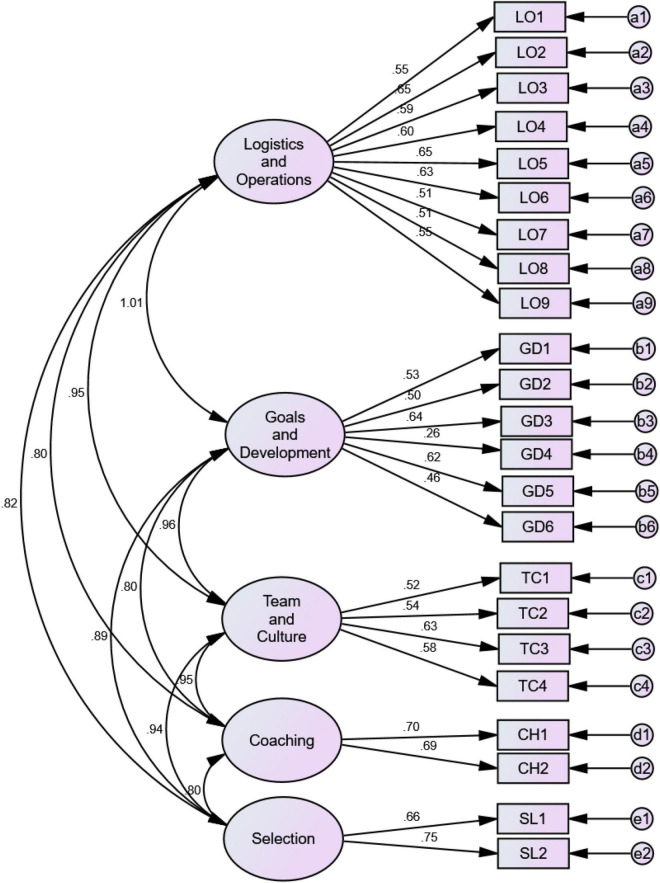
Multi-factor first-order CFA model with the original 23-items.

The study first sought to assess the convergence validity and reliability of the OSI-SP scale in the Ghanaian context. The result for the convergent validity is shown in Figure 1 and Table 1.

**TABLE 1:** Factor loadings, AVE, and CR of the original scale.

SN	Sample of behaviors	Factor loadings	*p*-value	AVE	CR
	**Logistics and operations**	–		0.34	0.82
LO1	The regulations in my sport	0.55	0.000*		
LO2	The accommodation used for training or competitions	0.65	0.000*		
LO3	The training or competition venue	0.59	0.000*		
LO4	The organization that governs and controls my sport	0.60	0.000*		
LO5	What gets said or written about me in the media	0.65	0.000*		
LO6	The organization of the competitions that I perform in	0.63	0.000*		
LO7	The funding allocations in my sport	0.51	0.000*		
LO8	The technology used in my sport	0.51	0.000*		
LO9	Traveling to or from training or competitions	0.55	0.000*		

	**Goals and development**	–		0.27	0.67
GD1	The spectators that watch me perform	0.53	0.000*		
GD2	The food that I eat	0.50**	0.000*		
GD3	My training schedule	0.64	0.000*		
GD4	Injuries	0.26**	0.000*		
GD5	The development of my sporting career	0.62	0.000*		
GD6	My goals	0.46**	0.000*		

	**Team and culture**	–		0.33	0.65
TC1	The responsibilities that I have on my team	0.52	0.000*		
TC2	The atmosphere surrounding my team	0.54	0.000*		
TC3	My teammates/other officials’ attitudes	0.63	0.000*		
TC4	The shared beliefs of my teammates/other officials	0.58	0.000*		

	**Coaching**	–		0.48	0.65
CH1	The relationship between my coach and I	0.70	0.000*		
CH2	My coach’s personality	0.69	0.000*		

	**Selection**	–		0.50	0.67
SL1	How my team is selected	0.66	0.000*		
SL2	Selection of my team for competition	0.75	0.000*		

*Significant at p < 0.001; **Items with weak loadings. AVE, average variance extracted; CR, composite reliability.

The results, as shown in Table 1, highlight the factor loadings (with associated *p*-values) of items, AVEs and the composite reliability of the dimensions. Regarding the logistics and operations dimension, loadings ranged from 0.51 to 0.65, that of goals and development ranged between 0.26 to 0.64, and team and culture had loadings within 0.52 to 0.63. The coaching dimension yielded loadings from 0.69 to 0.70 and the selection sub-scale showed loadings between 0.66 to 0.75. The standard criteria in structural equation modeling (SEM) for judging whether an item contributes significantly to a specific construct requires that factor loadings of items should be greater than 0.50 (Civelek, 2018). Out of the 23-items on the original scale, 20 met this condition, suggesting some level of convergence validity (even though the loadings were not so large). Surprisingly, all the three items which failed to meet the 0.50 standard were under the goals and development dimension.

Another criterion for evaluating the convergent validity of a scale is the use of AVE. The AVE describes the amount of variances a set of items contributes to a construct or a sub-dimension of a scale. Ideally, the AVE of the scale should be 0.5 or greater (Fornell and Larcker, 1981). With regards to the results in Table 1, only the selection dimension met the criterion with AVE value of 0.50. The other four dimensions did not show a sufficient level of convergent validity with respect to the AVE criterion with a value of 0.27; this is because three items under this dimension displayed low factor loadings. Fornell and Larcker (1981) argued that the AVE procedure is quite stringent, thus, the values of AVE should be compared with the composite reliability. The condition Fornell and Larcker gave was that in cases where the AVE is less than 0.50 and the composite reliability is greater than 0.60, convergent validity is established to some degree. All the five dimensions had composite reliability estimates greater than 0.60 with logistics and operations having the highest reliability estimate of 0.82, implying an appreciable level of scale reliability (Karagöz, 2016).

After the removal of the three items, the factor loadings for the items did not change much. That is, the factor loadings for the items were approximately the same for the two models. This also translates into equivalence in terms of the AVEs. The details are shown in the Supplementary Appendix Figure 1.

### Discriminant Validity

The study also sought to examine the discriminant validity of the OSI-SP scale to understand the applicability of its factor structure. The results of the discriminant validity analysis are shown in Table 2.

**TABLE 2:** Heterotrait-monotrait (HTMT) ratio of correlation.

S/N	Sub-scale (23-items)	GD	LO	TC	CH	SL
GD	Goals and development	1				
LO	Logistics and operations	0.81	1			
TC	Team and culture	0.88	**0.96**	1		
CH	Coaching	0.87	**0.94**	**0.93**	1	
SL	Selection	0.89	**0.93**	**0.92**	0.81	1

	**Sub-scales (20-items)**	**GD**	**LO**	**TC**	**CH**	**SL**

GD	Goals and development	1				
LO	Logistics and operations	0.80	1			
TC	Team and culture	0.86	0.85	1		
CH	Coaching	0.88	0.83	**0.91**	1	
SL	Selection	0.87	0.84	**0.90**	**0.91**	1

*In bold: Correlation ratios which failed to meet the 0.90 HTMT criterion.*

In assessing the discriminant validity of the OSI-SP scale, we used the HTMT 0.90 criterion, which requires that the correlation ratios of the dimensions should be less than 0.90 (Hensler et al., 2015). Based on the results in Table 2, 50% of the correlation ratios met the criterion for the original 23-item instrument. That is, whereas 5 coefficients of the correlation ratios were less than 0.90, the other 5 were greater than 0.90. This result suggests that the discriminant validity of the scale is not sufficient.

We explored the possible cause of the poor model fitting in the first model specified by removing the three items (GD2, GD4, GD6) under the goals and development dimension. The HTMT results for the 20-item model showed improvement in the discriminant validity indicators as compared to the 23-item model. The results of the HTMT correlation ratio in Table 2 further revealed that three of the correlations violated the discriminant validity criterion based on Hensler et al.’s (2015) condition (i.e., the HMTM correlation ratio between any two sub-scales should be less than 0.90). However, this cannot be assumed to be conclusive regarding the discriminant validity. More needs to be done to achieve a complete level of discriminant validity.

### Model Fit Indices for the Measurement Model

To test whether the hypothesized model fits the data collected using a sample of sport performers in Ghana, the model fit indices were assessed. Further, the new model (with the 20-items) was then compared with the first model (with 23-items) using the AIC value and other model fit indicators (Civelek, 2018). For the evaluation of the model fit indices for the models, the following cut-off points were utilized: Chi-square (*p* > 0.05; Civelek, 2018), CMIN/DF (<3.0; Schumacker and Lomax, 2004), GFI (>0.95; Mueller and Hancock, 2004), AGFI (>0.95; Civelek, 2018), RMSEA (<0.07; Hair et al., 2016), CFI (>0.95; Mueller and Hancock, 2004), TLI (>0.95; Schumacker and Lomax, 2004) and AIC (least values approximate reality). The model fit indices are shown in Table 3.

**TABLE 3:** Model fit indices.

Fit indices	Values(23-items)	Values(20-items)
Chi-square (χ^2^)	663.91, *p* < 0.001	517.829, *p* < 0.001
CMIN/DF	3.018	3.236
Goodness-of-Fit (GFI)	0.873	0.883
Adjusted Goodness-of-Fit (AGFI)	0.841	0.847
Root Mean Square Error of Approximation (RMSEA)	0.069	0.073
Comparative Fit Index (CFI)	0.863	0.878
Tucker-Lewis Index (TLI)	0.843	0.878
Akaike Information Criterion (AIC)	775.906	617.829

As presented in Table 3, almost all the model fit indicators for the 23-item model showed that the data did not fit the hypothesized model. This could be as a result of model misspecification, or perhaps, lack of discriminant validity (Kline, 2011). Based on the criteria by Hair et al. (2016), it was only the RMSEA fit indicator that showed a good fit with a very close value of 0.069.

The model fit indices for the modified model (20-items) appeared better than the original model with 23-items (Table 3). The AIC index for the 23-item model was 775.906 and that of the 20-item model was 617.829 indicating that the second model (20-item) is the model which is closer to reality and demonstrates sufficient fit (Civelek, 2018). Although the 20-item model showed adequate fit as compared to the 23-item model, the model fit indices for the model (20-item) failed to meet the standards by the selected scholars. The implications of the results are discussed in the next section.

## Discussion

The cross-cultural validation of OSI-SP is still at the infancy stage, although its use is widespread (see Olusoga, 2011; Arnold et al., 2016, 2017b). This study adds to the knowledge of the cross-cultural applicability of the OSI-SP instrument in Ghana. Our investigation revealed that the extent of the validity of the OSI-SP scale in the Ghanaian context is limited. Whereas some of the indicators from the analysis showed a sufficient level of validity, other indices showed otherwise. For example, the standardized factor loadings of all the items met the 0.50 criterion, except for three of them. Notably, it was evident that the logistics and operations, team and culture, coaching, and selection dimensions all showed acceptable fit. However, the goals and development dimension did not, a finding that is consistent with other research (see Arnold et al., 2017b). Possible reasons that might have accounted for this finding could be variations in culture and contextual norms (Arnold et al., 2017b; Liu et al., 2018).

The AVEs, on the other hand, were poor for four of the sub-dimensions with only one (i.e., selection dimension) satisfying the 0.50 criterion. Also, the majority of the model fit indices deviated from the criteria set, although these estimated indices were close to the standard criteria. It must be mentioned that these indicators for judging the quality of items and instruments should not be taken as golden rules, as there are disagreements over them in the statistics literature (Heene et al., 2011). Taking CFI, for example, Bentler (1992) proposed a value greater than 0.90, Hu and Bentler (1999) opted for a cut-off value near 0.95. Mueller and Hancock (2004) also recommended a value greater than 0.95. For Williams et al. (2009), several statistical values (such as factor loadings, significant values, standardized residuals, reliability coefficients) should be considered before a conclusion can be made regarding the quality of the items and/or scale. Marsh et al. (2004) advised that cut-off values for the validation should be used as a guide instead of using them as absolute values. Therefore, the findings of this study were operationalized based on the recommendations of William et al. and Marsh et al. that, the OSI-SP had modest applicability in the Ghanaian cultural setting. This, however, calls for a close relook at the items and the dimensions of the instrument by future researchers.

The current study is necessary as the cultural applicability of the OSI-SP appears to be still ongoing (Arnold et al., 2013). Other studies have tested the cultural validity of the OSI-SP instrument; while some have had results that are consistent with that of this research, others have found different results. Consistent with this study, Liu et al. (2018) found that the OSI-SP scale had limited applicability to the Taiwan setting. Similar findings were found in the Chinese settings that the OSI-SP scale did not have optimum cross-cultural validity (Arnold et al., 2017b). Among Malaysian and British samples, however, the OSI-SP indicators were found to be applicable and thus, provided evidence of cultural validity of the instrument (Arnold et al., 2016). For the British sample, in particular, the trend of results is not surprising since the original instrument was developed in the British setting. Although not certain, the Malaysian sample could have similar characteristics as the sample used in the development and validation of the original scale by Arnold et al., 2013. The similarities and discrepancies in the findings of this research against previous studies can largely be explained by cultural differences and sample characteristics. It is quite obvious that the culture of Ghana would be different from that of Britain, Malaysia, China and Taiwan, and this cultural disparity may penetrate through all aspects of the lives of the people (see Atkinson, 1985; Atkinson and Gim, 1989; Sun et al., 2004). With regards to the sample characteristics, the studies conducted in Britain, Malaysia, Taiwan and China sampled athletes with heterogeneous cultural characteristics (e.g., individualist-collectivist and different linguistic backgrounds) and from varied sport backgrounds (Arnold et al., 2013) whereas the majority of the sample in this research came from the non-elite football population (i.e., from a collectivist culture) in Ghana with varied educational level. That is, the majority of the soccer players used in this study had not schooled up to the tertiary level so there could be linguistic challenges with interpreting the English items on the OSI-SP instrument although thorough briefings and interpretations were done by the members of the research team before the completion of the questionnaire. Further, from the perception of competence perspective, late identification and career pursuits of most football players of African origin who ply in the local leagues compared to their counterparts from western societies (Charitas, 2017), are likely to develop low perceptions of competence, not clearly aligned to their developmental domains (i.e., physical, technical, psychological). Consequently, these players are unable to differentiate between effort, ability, and task difficulties within their competitive environment (Horn, 2015). These observations could potentially explain why the OSI-SP had low applicability in Ghana since the original instrument was developed using educated athletes.

Specifically, for the lower alphas reported for the goals and development subscale with the Ghanaian football participants, one key characteristic between individualistic and collectivistic societies is whether personal or group goals have priority. This perspective could possibly explain why some of the goal-related items on the OSI-SP developed and initially validated in an individualist culture like England are not as applicable to a collectivist society like Ghana (Triandis, 1995). We propose that future studies re-examine the appropriateness of the goals and development items to other Ghanaian samples. It is possible that some specific cultural peculiarities (e.g., linguistic variations) and norms need to be considered when investigating the organizational pressures among Ghanaian samples, compared to western samples in sport across specific issues on the goals and development stressors such as the food available to them, the development of a sporting career (e.g., early versus late sport specialization), and training schedules (Si et al., 2011, 2015; Lu, 2014; Tian et al., 2015). These highlighted examples could be peculiar stressors experienced by sport performers operating in the Ghanaian context. Perhaps, additional testing of the cross-cultural validity of the OSI-SP by translating the original instrument into a common Ghanaian language and other languages in the African contexts might help minimize the discrepancy identified in the current study.

### Strength and Limitations

The use of an advance analytical strategy (i.e., SEM) provides much stronger cross-cultural empirical evidence in the applicability of the OSI-SP. However, despite this strength, the study participants were all recruited from only one sporting discipline (i.e., football) in Ghana. Customarily, Ghana is a collectivist society. Hence, cultural beliefs might influence athletes’ behavior in sports compared to individualistic or mixed societies. Besides, because the sample of this study was drawn from only professional footballers, whether identified results can be generalized to professional athletes in other sporting disciplines in Ghana require further examination. The study did not focus on ethnic, language, cultural and religious backgrounds of respondents which might have influenced the findings. Moreover, the study failed to employ measurement invariance and differential analysis procedures which pose significant threats to the findings. Therefore, making generalized conclusions with such data may not be appropriate.

## Conclusion and Practical Implications

In this study, we assessed the cross-cultural validity of the OSI-SP questionnaire among Ghanaian sports persons, particularly football players (males) in the 2020/2021 GPL season. The original scale did not show a high level of cultural applicability in the Ghanaian context. However, after three items with low loadings were deleted, the 20-item scale showed an improved level of validity in terms of model fit indices over the 23-item scale. This does not suggest that the original scale should not be adopted and utilized in Ghana but the information from this study should rather be used as a starting point to understand the cultural validity of the OSI-SP in Ghana. The findings of this study have important practical implications that could offer support for the external validity and cross-cultural applicability of the OSI-SP instrument in measuring organizational stressors in sport. Most importantly, sport psychologists and coaches working closely with these footballers can now use the indicator to assess the organizational demands associated with the GPL.

Researchers who would adopt the OSI-SP questionnaire for their research in Ghana should carry out re-validation and confirmation of the applicability of the scale to the setting (in terms of the characteristics [e.g., religion, language, ethnicity, other cultural characteristics] of the participants, sports type) before major statistical analyses are carried out. More studies on organizational stressors are required, not only for the quest for knowledge but also for the promotion of sport performers’ psychological well-being. In future, scholars should conduct re-validation of OSI-SP to establish its applicability, focusing on other sport disciplines, gender, religion, among others, using measurement invariance and differential analysis procedures.

## Data Availability Statement

The raw data supporting the conclusions of this article will be made available by the authors, without undue reservation.

## Ethics Statement

The study involved human participants and Ethical approval was granted from the Institutional Review Board of the University of Cape Coast to conduct the study with reference number UCC/IRB/A/2016/794. The patients/participants provided their written informed consent to participate in this study.

## Author Contributions

MS-S and JH conceived the idea. FQ performed the analysis. MS-S, FQ, JF, JH, and TS prepared the initial draft of the manuscript. All authors thoroughly revised and approved the final version of the manuscript.

## Conflict of Interest

The authors declare that the research was conducted in the absence of any commercial or financial relationships that could be construed as a potential conflict of interest.

## Publisher’s Note

All claims expressed in this article are solely those of the authors and do not necessarily represent those of their affiliated organizations, or those of the publisher, the editors and the reviewers. Any product that may be evaluated in this article, or claim that may be made by its manufacturer, is not guaranteed or endorsed by the publisher.
