# Supplementary material for: Cross-Cultural Applicability of Organizational Stressor Indicator for Sport Performers Questionnaire in Ghana Using Structural Equation Modeling Approach
Source: Front Psychol. 2021 Dec 13;12:772184. doi: 10.3389/fpsyg.2021.772184 (PMC8710532; doi:10.3389/fpsyg.2021.772184)
Supplement: Supplementary file 1 [file Image_1.pdf]

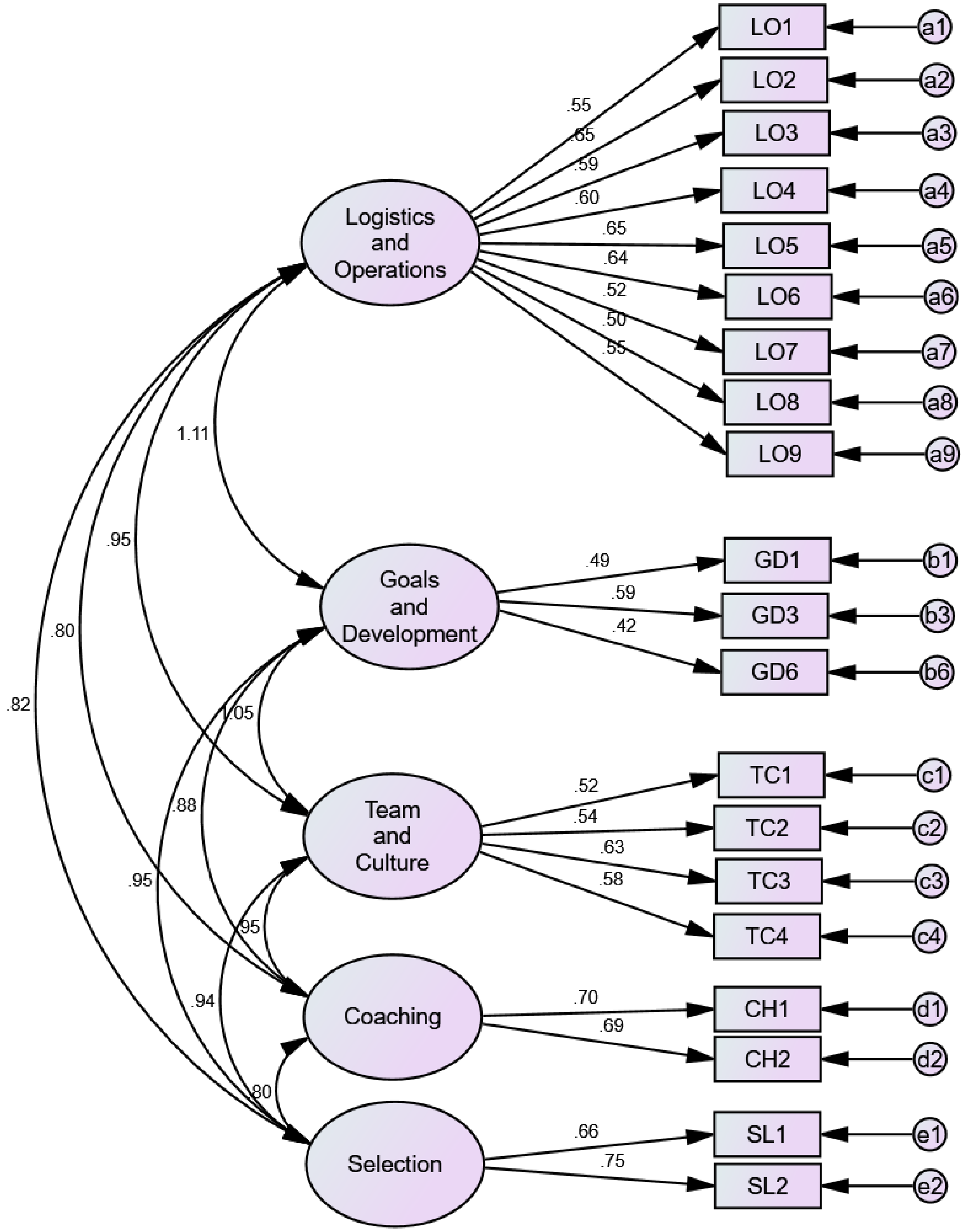

Logistics and Operations
Goals and Development
Team and Culture
Coaching
Selection
LO1
LO2
LO3
LO4
LO5
LO6
LO7
LO8
LO9
GD1
GD3
GD6
TC1
TC2
TC3
TC4
CH1
CH2
SL1
SL2
a1
a2
a3
a4
a5
a6
a7
a8
a9
b1
b3
b6
c1
c2
c3
c4
d1
d2
e1
e2
.55
.65
.59
.60
.65
.64
.52
.50
.55
.49
.59
.42
.52
.54
.63
.58
.70
.69
.66
.75
1.11
.95
.80
.82
1.05
.88
.95
.95
.94
.80

Standardized Regression Weights: (Group number 1 - Default model)

			Estimate
LO1	<---	LaO	.538
LO2	<---	LaO	.650
LO3	<---	LaO	.593
LO4	<---	LaO	.606
LO5	<---	LaO	.650
LO6	<---	LaO	.636
LO7	<---	LaO	.518
LO8	<---	LaO	.509
LO9	<---	LaO	.548
GD1	<---	GaD	.530
GD3	<---	GaD	.654
GD5	<---	GaD	.617
TC1	<---	TaC	.518
TC2	<---	TaC	.539
TC3	<---	TaC	.628
TC4	<---	TaC	.580
CH1	<---	CHG	.701
CH2	<---	CHG	.690
SL1	<---	SEL	.661
SL2	<---	SEL	.754
